# Factors associated with the efficacy of smoking cessation treatments and predictors of smoking abstinence in EAGLES

**DOI:** 10.1111/add.14208

**Published:** 2018-03-30

**Authors:** Robert West, A. Eden Evins, Neal L. Benowitz, Cristina Russ, Thomas McRae, David Lawrence, Lisa St Aubin, Alok Krishen, Melissa C. Maravic, Robert M. Anthenelli

**Affiliations:** ^1^ University College London London UK; ^2^ Massachusetts General Hospital and Harvard Medical School Boston MA USA; ^3^ University of California San Francisco CA USA; ^4^ Pfizer New York NY USA; ^5^ PAREXEL International on behalf of GSK, Research Triangle Park NC USA; ^6^ University of California San Diego CA USA

**Keywords:** Bupropion, nicotine replacement therapy, placebo, smoking cessation, treatment effects, varenicline

## Abstract

**Aims:**

To assess (1) how far the efficacies of front‐line smoking cessation pharmacotherapies vary as a function of smoker characteristics and (2) associations between these characteristics and success of smoking cessation attempts.

**Design:**

Prospective correlational study in the context of a double‐blind randomized trial. The outcome was regressed individually onto each covariate after adjusting for treatment, and then a forward stepwise model constructed. Treatment moderator effects of covariates were tested by treatment × covariate interactions.

**Setting:**

Health service facilities in multiple countries.

**Participants:**

Data came from 8120 smokers willing to make a quit attempt, randomized to varenicline, bupropion, nicotine replacement therapy (NRT) or placebo in Evaluating Adverse Events in a Global Smoking Cessation Study (EAGLES) between 30 November 2011 and 13 January 2015.

**Measurements:**

Smoker characteristics measured at baseline were country, psychiatric history, sex, age, body mass index (BMI), ethnic group, life‐time suicidal ideation/behaviour, anxiety, depression, aggression, psychotropic medication, history of alcohol/substance use disorder, age of starting smoking, cigarette dependence [Fagerström Test for Cigarette Dependence (FTCD)] and prior use of study medicines. Outcome was biochemically confirmed continuous abstinence at weeks 9–24 from start of treatment.

**Findings:**

No statistically significant treatment × covariate interactions were found. Odds of success were associated independently positively with age [odds ratio (OR) = 1.01; 95% confidence interval (CI) = 1.00, 1.01], BMI (1.01; 95% CI = 1.00, 1.02) and age of starting smoking (1.03; 95% CI = 1.02, 1.04). Odds were associated independently negatively with US (versus non‐US) study site (0.53; 95% CI = 0.46, 0.61), black (versus white) ethnic group (0.57; 95% CI = 0.45, 0.72), mood disorder (0.85; 95% CI = 0.73, 0.99), anxiety disorder (0.71; 95% CI = 0.55, 0.90) and psychotic disorder (0.73; 95% CI = 0.50, 1.07), taking psychotropic medication (0.81; 95% CI = 0.68, 0.95), FTCD (0.89; 95% CI = 0.87, 0.92) and previous use of NRT (0.78; 95% CI = 0.67, 0.91).

**Conclusions:**

While a range of smoker characteristics—including psychiatric history, cigarette dependence and prior use of nicotine replacement therapy (NRT)—are associated with lower cessation rates, they do not substantially influence the efficacy of varenicline, bupropion or NRT.

## Introduction

Evaluating Adverse Events in a Global Smoking Cessation Study (EAGLES; NCT01456936) was a large, multi‐centre, randomized, placebo‐controlled trial that examined the safety and efficacy of varenicline, bupropion and transdermal nicotine patch [a form of nicotine replacement therapy (NRT)] for smoking cessation 1. The size of the trial, randomizing smokers in a triple‐dummy fashion into three active treatments and placebo, including smokers from multiple countries with varying psychiatric histories, and gathering data on a wide range of smoker characteristics provided an unparalleled opportunity to assess how smoker characteristics predict smoking cessation outcomes and how they may influence treatment efficacy.

It is important to accumulate information on smoker characteristics that influence the success of stopping smoking. Evidence suggests that older age and markers of lower cigarette dependence are predictive of success 2, 3, 4, 5, 6, although not all studies show these results 7. There is less consistent, conflicting or negative evidence on the effects of age of smoking onset 2, 3, substance use disorder 8, psychiatric disorder 6, 9, gender 4, 6, 10, geographical location 11, 12, body mass index (BMI) 2, 3, 10, 13, 14 and prior use of smoking cessation treatments 15. These covariates were assessed in the EAGLES trial. It was reported in the main outcome paper that psychiatric history (versus no history) was associated with lower success rates and that this variable did not interact with treatment efficacy 1. However, the predictive effects of other variables assessed have not been reported. With such a large and diverse sample receiving a variety of treatments and a substantial number receiving placebo, the EAGLES trial can add significantly to our knowledge to help build models of cigarette addiction and assist clinicians in treating this disorder.

Clinically, it is important to know how treatment efficacy varies as a function of smoker characteristics. Moderators of treatment efficacy have not been studied widely in randomized clinical trials (RCTs), but some evidence is emerging. A recent meta‐analysis found that varenicline was more effective for female than for male smokers for short‐ to medium‐term cessation (up to 6 months) 16. Smokers with a faster rate of nicotine metabolism appear to be less successful when using nicotine patches than those with a slower metabolism 17. A placebo‐controlled RCT of nicotine nasal spray appeared to show greater efficacy in more addicted smokers 18. One study found that smokers with a psychiatric disorder benefited more from ‘dual form’ NRT (transdermal patch plus a faster‐acting product) versus no NRT than those without a psychiatric disorder 19. A study of methylphenidate to aid smoking cessation in smokers with attention deficit hyperactivity disorder reported in a *post‐hoc* analysis an effect only in those smokers with more severe symptoms 20. A *post‐hoc* analysis of two RCTs of NRT found reduced efficacy in overweight women 21. It has been proposed that female smokers may not respond as well to NRT or bupropion as male smokers 22. A genome‐wide association study failed to identify specific associations with NRT treatment efficacy, but the results were reported to be suggestive of specific regions that merit further scrutiny 23.

On common sense grounds, one may hypothesize that smokers who have used a given form of treatment in the past may benefit less from that treatment because their past failure is indicative of treatment resistance. However, a placebo‐controlled RCT of varenicline in smokers who had used this drug previously found effect sizes similar to those in a study of drug‐naive smokers 15.

Very little research has been undertaken on smoker characteristics that may moderate relative effectiveness of different forms of treatment compared with each other. In clinical practice in the United Kingdom, the relative efficacy of varenicline and NRT has been found to vary throughout different local services 24, but the service characteristics that underlie this are not known. Some evidence suggests that varenicline is more effective than NRT in smokers with ‘normal’ rates of nicotine metabolism, but not in those who metabolize nicotine slowly 25. However, the relative efficacy of different pharmacotherapies as a function of other phenotypes has not been studied. Such research is difficult because it requires large sample sizes and randomization to multiple active treatments.

This study aimed to assess (1) how far the efficacy of the pharmacotherapies tested varied as a function of smoker characteristics and (2) associations of those characteristics with odds of smoking abstinence taking account of pharmacotherapy.

## Methods

### Design

EAGLES was a randomized (1 : 1 : 1 : 1), double‐blind, triple‐dummy, placebo‐ and active‐ (nicotine patch, NRT; 21 mg/day with taper) controlled trial of varenicline (1 mg twice daily) and bupropion (150 mg twice daily) for 12 weeks with 12‐week non‐treatment follow‐up. All subjects received repeated brief smoking cessation counselling. The published trial report provides full details of the study design and efficacy outcome measures, and continuous abstinence at the end of treatment (weeks 9–12) and to the end of the study (weeks 9–24) 1. The trial protocol is registered with http://clinicaltrials.gov (NCT01456936).

This paper reports an exploratory analysis to assess predictors of abstinence and interactions between study drug and smoker variables on carbon monoxide‐verified smoking abstinence from weeks 9 to 24 after treatment initiation. We used weeks 9–24 (a period that includes the last 4 weeks of treatment and 12‐week non‐treatment follow‐up) as this provided a measure that is closest to the Russell Standard, the recommended end‐point to ensure greatest confidence in extrapolating to life‐time abstinence 26. Following the Russell Standard, those failing to record primary outcome data were classified as continuing smokers.

### Participants

Participants in EAGLES were male and female smokers, aged 18–75 years, with and without pre‐specified psychiatric diagnoses who smoked an average of ≥ 10 cigarettes/day. They were recruited from 16 countries. A list of participating study sites was published previously 1. Supporting information, Table S1 details subject enrolment and site presence by country/region by treatment.

The sample included smokers with psychotic, anxiety and mood disorders meeting the *Diagnostic and Statistical Manual of Mental Disorders,* 4th edn, text revision diagnostic criteria confirmed by Structured Clinical Interview for *Diagnostic and Statistical Manual of Mental Disorders*, Axis I and Axis II disorders (SCID‐I and SCID‐II) 27, without clinical exacerbation in the prior 6 months, with stable treatment (medication and dose) for ≥ 3 months (if on treatment) and not judged to be at imminent risk of self‐injurious or suicidal behaviour.

A total of 8120 subjects were included in the present analyses (varenicline, *n* = 2030; bupropion, *n* = 2029; NRT, *n* = 2029; placebo, *n* = 2032). Twenty‐four participants with borderline personality disorder were excluded because the small size of this group did not permit accurate modelling involving this disorder. The sample was not powered to detect interactions between treatment group and smoker characteristics, and the analyses were not pre‐planned and thus should be considered exploratory.

### Smoker characteristics

The smoker characteristics examined were: country of study site (USA versus non‐USA); history of psychiatric diagnosis (defined as the primary diagnosis: none, mood disorder, anxiety disorder, psychotic disorder); sex; age; BMI; ethnic group (white, black, other); lifetime suicidal ideation and/or behaviour [none versus any, captured through Columbia–Suicide Severity Rating Scale (C‐SSRS)] 28; anxiety score [Hospital Anxiety and Depression scale (HADS)] 29; depression score (HADS) 29; aggression score [Buss–Perry Aggression Questionnaire (BPAQ)] 30; use of psychotropic medication, including sleeping aids (none, any); cigarette dependence [Fagerström Test for Cigarette Dependence (FTCD)] 31; age of starting smoking; and prior use of study medicines (none versus any, for each of the three medications—varenicline, bupropion or any type of NRT). Supporting information, Table S2 describes the collection of smokers’ characteristics as recorded on the patient report form used by investigators.

### Statistical analyses

The data analysis involved a series of logistic regressions, with continuous abstinence from weeks 9 to 24 as the outcome measure. The analyses did not include study site as a level or factor because of the large number of sites with many contributing no participants. The effect of each individual covariate was studied using logistic regression controlling for the treatment effect, while also including the treatment × covariate interaction (model 1). As this model does not allow for potential collinearity between covariates, all covariates were entered into a forward stepwise logistic regression model that controlled for the treatment effect, retaining a covariate until no significant improvement in the model fit was seen using a threshold of *P* = 0.05 (model 2). Additional analyses were performed by categorizing the numerical covariates in model 1 to facilitate the interpretation of these covariate effects.

## Results

Table 1 shows the sample characteristics of the four treatment groups and their similarities.

**Table 1:** Sample characteristics.

	Varenicline	Bupropion	NRT	Placebo	All
Covariate	(n = 2030)	(n = 2029)	(n = 2029)	(n = 2032)	(n = 8120)
Age, years	46.53 (12.35)	46.28 (12.63)	46.87 (12.14)	46.43 (12.17)	46.53 (12.33)
Age of starting smoking, years	18.16 (5.77)	18.10 (5.95)	18.31 (6.06)	18.20 (5.99)	18.19 (5.94)
Female	1118 (55.1%)	1128 (55.6%)	1143 (56.3%)	1147 (56.4%)	4536 (55.9%)
Ethnic group					
White	1676 (82.6%)	1655 (81.6%)	1640 (80.8%)	1655 (81.4%)	6626 (81.6%)
Black	284 (14.0%)	285 (14.0%)	309 (15.2%)	283 (13.9%)	1161 (14.3%)
Other	70 (3.4%)	89 (4.4%)	80 (3.9%)	93 (4.6%)	332 (4.1%)
Country					
USA	1065 (52.5%)	1067 (52.6%)	1061 (52.3%)	1065 (52.4%)	4258 (52.4%)
Non‐USA	965 (47.5%)	962 (47.4%)	968 (47.7%)	967 (47.6%)	3862 (47.6%)
BMI, kg/m^2^	28.13 (6.41)	28.13 (6.43)	27.98 (6.29)	28.28 (6.39)	28.13 (6.38)
History of psychiatric diagnosis					
None	1005 (49.5%)	1001 (49.3%)	1013 (49.9%)	1009 (49.7%)	4028 (49.6%)
Mood disorder	734 (36.2%)	729 (35.9%)	721 (35.5%)	726 (35.7%)	2910 (35.8%)
Anxiety disorder	196 (9.7%)	201 (9.9%)	196 (9.7%)	199 (9.8%)	792 (9.8%)
Psychotic disorder	95 (4.7%)	98 (4.8%)	99 (4.9%)	98 (4.8%)	390 (4.8%)
Alcohol/substance use disorder history	228 (11.2%)	243 (12.0%)	235 (11.6%)	245 (12.1%)	951 (11.7%)
HADS anxiety score	3.93 (3.52)	4.00 (3.67)	3.94 (3.57)	4.05 (3.49)	3.98 (3.56)
HADS depression score	2.36 (2.89)	2.44 (3.02)	2.34 (2.85)	2.35 (2.76)	2.37 (2.88)
C‐SSRS prior suicidal ideation and/or behaviour	400 (19.7%)	409 (20.2%)	388 (19.1%)	411 (20.2%)	1608 (19.8%)
BPAQ aggression score	54.75 (16.93)	55.26 (17.41)	56.03 (17.78)	55.58 (17.18)	55.40 (17.33)
Baseline psychotropic medication	687 (33.8%)	629 (31.0%)	647 (31.9%)	654 (32.2%)	2617 (32.2%)
FTCD	5.77 (1.97)	5.79 (1.99)	5.76 (1.97)	5.71 (2.02)	5.76 (1.99)
Prior varenicline use	286 (14.1%)	348 (17.2%)	331 (16.3%)	306 (15.1%)	1271 (15.7%)
Prior bupropion use	211 (10.4%)	221 (10.9%)	206 (10.2%)	206 (10.1%)	844 (10.4%)
Prior any NRT use	523 (25.8%)	539 (26.6%)	544 (26.8%)	526 (25.9%)	2132 (26.3%)

NRT = nicotine replacement therapy (transdermal nicotine patch); BMI = body mass index; HADS = Hospital Anxiety and Depression Scale; C‐SSRS = Columbia‐Suicide Severity Rating Scale; BPAQ = Buss–Perry Aggression Questionnaire; FTCD = Fagerström Test for Cigarette Dependence; SD = standard deviation. Data are mean (SD) or *n* (%).

The odds ratios (ORs) for active treatment versus placebo, the results of the model 1 analysis for all covariates entered singly and the results of the stepwise model 2 analysis are shown in Table 2.

**Table 2:** Predictors and moderators of abstinence during weeks 9–24 (univariate analysis, model 1 and stepwise regression analyses, model 2).

	Model 1 (univariate)^a^	P‐value for treatment × covariate interaction (testing for treatment moderation)	Model 2 (stepwise)^b^
Treatment			
Varenicline versus placebo	2.70 (2.25–3.25)	–	2.84 (2.35–3.42)
Bupropion versus placebo	1.88 (1.56–2.28)	–	1.96 (1.61–2.38)
NRT versus placebo	1.81 (1.50–2.20)	–	1.86 (1.53–2.27)
Covariate			
Country	–	0.8235	–
USA versus non‐USA	0.48 (0.42–0.55)	–	0.53 (0.46–0.61)
Psychiatric diagnosis	–	0.6290	–
Mood disorder versus no diagnosis	0.73 (0.64–0.84)	–	0.85 (0.73–0.99)
Anxiety disorder versus no diagnosis	0.61 (0.48–0.78)	–	0.71 (0.55–0.90)
Psychotic disorder versus no diagnosis	0.48 (0.33–0.70)	–	0.73 (0.50–1.07)
Age, years	–	0.1756	–
1‐year increase	1.01 (1.00–1.03)	–	1.01 (1.00–1.01)
Age of starting smoking, years	–	0.5624	–
1‐year increase	1.04 (1.02–1.06)	–	1.03 (1.02–1.04)
Gender	–	0.5794	Dropped
Female versus male	0.97 (0.85–1.10)	–	–
Ethnic group	–	0.3421	–
Black versus white	0.42 (0.33–0.53)	–	0.57 (0.45–0.72)
Other versus white	0.74 (0.52–1.06)	–	0.83 (0.60–1.15)
BMI, kg/m^2^	–	0.6027	–
1‐unit increase	0.99 (0.96–1.01)	–	1.01 (1.00–1.02)
Alcohol/substance use disorder history	–	0.8222	Dropped
Yes versus no	0.69 (0.55–0.85)	–	–
HADS anxiety score	–	0.3222	Dropped
1‐unit increase	0.93 (0.89–0.98)	–	–
HADS depression score	–	0.2031	Dropped
1‐unit increase	0.93 (0.88–0.99)	–	–
C‐SSRS prior suicidal ideation or behaviour	–	0.6296	Dropped
Yes versus no	0.74 (0.63–0.88)	–	–
BPAQ aggression score	–	0.0666	Dropped
1‐unit increase	1.00 (0.99–1.01)	–	–
Baseline psychotropic medication	–	0.2465	–
Yes versus no	0.68 (0.59–0.78)	–	0.81 (0.68–0.95)
FTCD	–	0.3703	–
1‐unit increase	0.85 (0.79–0.92)	–	0.89 (0.87–0.92)
Prior use of varenicline	–	0.6799	Dropped
Yes versus no	0.78 (0.64–0.93)	–	–
Prior use of bupropion	–	0.6226	Dropped
Yes versus no	0.80 (0.64–0.99)	–	–
Prior use of NRT	–	0.8185	–
Yes versus no	0.69 (0.59–0.80)	–	0.78 (0.67–0.91)

NRT = nicotine replacement therapy (transdermal nicotine patch; BMI = body mass index; HADS = Hospital Anxiety and Depression Scale; C‐SSRS = Columbia‐Suicide Severity Rating Scale; BPAQ = Buss–Perry Aggression Questionnaire; FTCD = Fagerström Test for Cigarette Dependence; OR = odds ratio; CI = confidence interval. Data are OR (95% CI), unless stated otherwise.

a Model 1: treatment included in model plus each other covariate on its own and the treatment × covariate interaction;

b model 2: final model included treatment plus other covariates added stepwise until no further significant improvement in fit.

The ORs for continuous abstinence weeks 9–24 for active treatment versus placebo based on the stepwise model were 2.84 [95% confidence interval (CI) = 2.35, 3.42] for varenicline, 1.96 (95% CI = 1.61, 2.38) for bupropion and 1.86 (95% CI = 1.53, 2.27) for NRT. From the stepwise model, abstinence was associated positively and independently with non‐US study site; higher age; higher BMI; white versus black race/ethnicity; not having a history of mood, anxiety or psychotic disorder; not taking psychotropic medication; lower cigarette dependence; not previously having used NRT; and higher age of starting smoking. Data in Table 2 also show that no treatment × covariate interactions added significantly to the prediction above the main effect for that covariate.

The percentages abstinent and the ORs stratified by covariate categories and treatment are presented in Table 3 (baseline demographic and smoking covariates) and Table 4 (baseline psychiatric history covariates).

**Table 3:** Percentage abstinent by treatment and ORs versus placebo for baseline demographic and smoking covariates (univariate analysis, model 1; categorical variables, with continuous variables transformed into categorical variables to aid interpretation).

	Varenicline		Bupropion		NRT		Placebo
Covariate	CAR weeks 9–24 % (95% CI)	versus placebo OR (95 % CI)	CAR weeks 9–24 % (95% CI)	versus placebo OR (95% CI)	CAR weeks 9–24 % (95% CI)	versus placebo OR (95 % CI)	CAR weeks 9–24 % (95% CI)
Region							
USA	16.1 (14.0–18.4)	2.66 (1.99–3.55)	11.4 (9.6–13.4)	1.78 (1.31–2.41)	10.6 (8.9–12.6)	1.64 (1.21–2.24)	6.7 (5.3–8.4)
Non‐USA	27.9 (25.2–30.8)	2.80 (2.20–3.55)	21.5 (19.0–24.2)	1.97 (1.54–2.52)	21.3 (18.9–24.0)	1.96 (1.53–2.50)	12.2 (10.2–14.4)
Age, years							
18–29	14.9 (10.9–20.0)	2.25 (1.21–4.18)	15.8 (11.7–21.0)	2.41 (1.30–4.46)	15.8 (11.4–21.5)	2.41 (1.28–4.54)	7.2 (4.4–11.4)
30–44	18.0 (15.1–21.4)	2.12 (1.50–2.98)	15.9 (13.2–19.0)	1.81 (1.29–2.56)	14.4 (11.8–17.5)	1.62 (1.14–2.31)	9.4 (7.3–11.9)
45–59	23.4 (20.8–26.3)	3.24 (2.45–4.30)	15.1 (12.8–17.7)	1.89 (1.40–2.55)	15.3 (13.0–17.8)	1.91 (1.42–2.57)	8.6 (6.9–10.6)
≥ 60	29.0 (24.2–34.4)	2.80 (1.85–4.25)	19.7 (15.7–24.3)	1.68 (1.09–2.58)	19.3 (15.4–23.9)	1.64 (1.06–2.53)	12.7 (9.4–16.9)
Age of starting smoking, years							
1 ≤ 16	17.8 (15.1–20.8)	3.43 (2.38–4.96)	13.0 (10.7–15.7)	2.38 (1.62–3.48)	12.5 (10.3–15.2)	2.28 (1.55–3.34)	5.9 (4.4–7.9)
≥ 16	23.8 (21.6–26.2)	2.50 (2.02–3.09)	17.8 (15.8–20.0)	1.73 (1.39–2.16)	17.4 (15.5–19.6)	1.69 (1.35–2.10)	11.1 (9.5–12.9)
Gender							
Female	22.5 (20.1–25.0)	2.89 (2.26–3.69)	15.6 (13.6–17.8)	1.83 (1.42–2.37)	15.3 (13.3–17.5)	1.79 (1.39–2.32)	9.1 (7.6–10.9)
Male	20.8 (18.3–23.5)	2.48 (1.88–3.26)	16.9 (14.6–19.5)	1.93 (1.45–2.56)	16.3 (14.0–18.9)	1.84 (1.38–2.45)	9.6 (7.8–11.7)
Ethnic group							
White	23.6 (21.6–25.7)	2.63 (2.17–3.19)	17.8 (16.0–19.7)	1.85 (1.51–2.26)	16.6 (14.9–18.5)	1.70 (1.39–2.08)	10.5 (9.1–12.0)
Black	11.2 (8.0–15.5)	2.87 (1.45–5.69)	7.3 (4.8–11.0)	1.80 (0.87–3.72)	10.0 (7.1–13.9)	2.52 (1.27–5.01)	4.2 (2.4–7.3)
Other	20.0 (12.2–30.9)	5.56 (1.74–17.75)	14.6 (8.6–23.5)	3.81 (1.19–12.16)	20.0 (12.6–30.1)	5.56 (1.78–17.42)	4.3 (1.6–10.9)
BMI, kg/m^2^							
< 25	20.0 (17.1–23.1)	2.25 (1.64–3.07)	17.5 (14.9–20.5)	1.91 (1.39–2.63)	15.7 (13.2–18.7)	1.68 (1.22–2.33)	10.0 (7.9–12.5)
25 ≤ 30	24.6 (21.5–28.0)	3.17 (2.32–4.32)	18.0 (15.2–21.0)	2.13 (1.54–2.94)	16.2 (13.6–19.1)	1.87 (1.35–2.60)	9.3 (7.3–11.7)
≥ 30	20.6 (17.6–23.9)	2.78 (2.00–3.88)	13.1 (10.7–16.0)	1.62 (1.14–2.32)	15.0 (12.4–18.0)	1.89 (1.33–2.67)	8.5 (6.6–10.9)
FTCD							
0–3	30.1 (24.9–35.8)	2.45 (1.63–3.69)	22.1 (17.6–27.4)	1.62 (1.06–2.48)	26.1 (21.2–31.7)	2.01 (1.32–3.05)	14.9 (11.3–19.4)
4–6	21.4 (18.9–24.0)	2.63 (2.03–3.43)	17.9 (15.6–20.4)	2.11 (1.61–2.77)	16.0 (13.9–18.4)	1.85 (1.41–2.43)	9.3 (7.6–11.3)
7–10	19.2 (16.5–22.1)	3.12 (2.24–4.36)	11.7 (9.7–14.2)	1.75 (1.23–2.49)	11.6 (9.5–14.1)	1.73 (1.21–2.48)	7.0 (5.4–9.1)
Prior varenicline use							
Yes	20.2 (16.0–25.3)	3.64 (2.13–6.22)	14.0 (10.8–18.1)	2.34 (1.36–4.04)	12.9 (9.7–17.0)	2.14 (1.23–3.72)	6.5 (4.2–9.9)
No	22.0 (20.1–24.0)	2.58 (2.13–3.14)	16.6 (14.9–18.5)	1.83 (1.49–2.24)	16.3 (14.6–18.1)	1.78 (1.45–2.19)	9.8 (8.5–11.3)
Prior bupropion use							
Yes	20.3 (15.4–26.3)	3.80 (1.98–7.31)	15.3 (11.2–20.7)	2.70 (1.38–5.28)	12.6 (8.7–17.8)	2.14 (1.07–4.30)	6.3 (3.6–10.5)
No	21.9 (20.0–23.8)	2.62 (2.16–3.17)	16.3 (14.6–18.0)	1.82 (1.49–2.22)	16.1 (14.5–17.8)	1.79 (1.47–2.19)	9.6 (8.4–11.1)
Prior NRT use							
Yes	18.5 (15.4–22.1)	3.10 (2.07–4.64)	12.2 (9.7–15.2)	1.90 (1.24–2.91)	12.5 (9.9–15.5)	1.94 (1.27–2.97)	6.8 (4.9–9.3)
No	22.8 (20.8–25.0)	2.61 (2.12–3.20)	17.6 (15.7–19.6)	1.88 (1.52–2.33)	16.9 (15.1–18.9)	1.79 (1.45–2.22)	10.2 (8.7–11.8)

CAR = continuous abstinence rate; CI = confidence interval; OR = odds ratio; NRT = nicotine replacement therapy (transdermal nicotine patch); BMI = body mass index; FTCD = Fagerström Test for Cigarette Dependence.

**Table 4:** Percentage abstinent by treatment and ORs versus placebo for baseline psychiatric history covariates (univariate analysis, model 1; categorical variables, with continuous variables transformed into categorical variables to aid interpretation).

	Varenicline		Bupropion		NRT		Placebo
Covariate	CAR weeks 9–24 % (95 % CI)	versus placebo OR (95% CI)	CAR weeks 9–24 % (95% CI)	versus placebo OR (95% CI)	CAR weeks 9–24 % (95% CI)	versus placebo OR (95% CI)	CAR weeks 9–24 % (95% CI)
Psychiatric diagnosis							
No diagnosis	25.4 (22.8–28.2)	2.91 (2.28–3.72)	18.7 (16.4–21.3)	1.97 (1.52–2.55)	18.4 (16.1–20.9)	1.93 (1.49–2.49)	10.5 (8.7–12.5)
Mood disorder	18.8 (16.1–21.7)	2.28 (1.67–3.11)	15.0 (12.6–17.8)	1.75 (1.27–2.41)	12.7 (10.5–15.3)	1.44 (1.03–2.01)	9.2 (7.3–11.5)
Anxiety disorder	16.3 (11.7–22.1)	2.79 (1.42–5.50)	10.4 (6.9–15.4)	1.67 (0.81–3.43)	15.8 (11.3–21.6)	2.69 (1.36–5.31)	6.5 (3.8–10.9)
Psychotic disorder	16.8 (10.5–25.7)	4.76 (1.53–14.82)	10.2 (5.5–17.9)	2.67 (0.81–8.83)	10.1 (5.5–17.7)	2.64 (0.80–8.72)	4.0 (1.5–10.3)
Alcohol/substance use disorder history							
Yes	15.3 (11.2–20.6)	2.16 (1.19–3.89)	11.1 (7.7–15.7)	1.49 (0.80–2.75)	12.3 (8.7–17.1)	1.67 (0.91–3.08)	7.7 (5.0–11.8)
No	22.5 (20.7–24.5)	2.76 (2.27–3.34)	16.9 (15.2–18.7)	1.92 (1.57–2.35)	16.2 (14.5–17.9)	1.83 (1.50–2.24)	9.5 (8.2–11.0)
HADS anxiety							
0–7	22.1 (20.2–24.2)	2.53 (2.08–3.07)	16.7 (15.0–18.5)	1.78 (1.46–2.18)	16.3 (14.6–18.1)	1.73 (1.41–2.12)	10.1 (8.7–11.6)
8–10	21.1 (16.2–27.1)	5.30 (2.60–10.82)	12.6 (8.7–17.8)	2.85 (1.34–6.07)	12.0 (8.2–17.3)	2.70 (1.26–5.81)	4.8 (2.6–8.7)
≥ 11	17.2 (11.4–25.2)	3.21 (1.30–7.93)	15.4 (10.2–22.5)	2.82 (1.15–6.89)	14.1 (8.9–21.6)	2.55 (1.01–6.39)	6.0 (2.9–12.2)
HADS depression							
0–7	21.7 (19.9–23.7)	2.59 (2.15–3.12)	16.3 (14.7–18.1)	1.82 (1.49–2.21)	15.8 (14.3–17.5)	1.75 (1.44–2.13)	9.7 (8.4–11.1)
8–10	23.8 (16.7–32.7)	10.02 (2.93–34.32)	18.0 (11.9–26.2)	7.03 (2.02–24.47)	9.1 (4.8–16.7)	3.24 (0.85–12.33)	3.0 (0.9–8.9)
≥ 11	15.3 (7.0–30.2)	2.45 (0.46–13.16)	7.4 (2.8–18.1)	1.08 (0.19–6.28)	26.1 (15.1–41.3)	4.79 (0.97–23.55)	6.8 (1.7–23.7)
C‐SSRS prior suicidal ideation or behaviour							
Yes	17.0 (13.6–21.0)	2.80 (1.76–4.46)	13.4 (10.4–17.1)	2.13 (1.32–3.43)	14.4 (11.2–18.2)	2.31 (1.43–3.72)	6.8 (4.7–9.6)
No	22.9 (20.9–25.0)	2.68 (2.20–3.27)	16.9 (15.1–18.8)	1.83 (1.49–2.26)	16.0 (14.3–17.9)	1.73 (1.40–2.13)	9.9 (8.6–11.5)
BPAQ aggression score							
< 60	22.2 (20.0–24.5)	2.75 (2.20–3.45)	18.2 (16.2–20.4)	2.15 (1.70–2.71)	16.1 (14.2–18.2)	1.85 (1.46–2.35)	9.4 (7.9–11.1)
≥ 60	20.9 (18.0–24.1)	2.59 (1.89–3.54)	12.3 (10.1–15.0)	1.38 (0.99–1.94)	15.1 (12.7–17.9)	1.75 (1.27–2.42)	9.2 (7.3–11.6)
Baseline psychotropic medication							
Yes	17.1 (14.5–20.1)	2.40 (1.70–3.39)	13.8 (11.3–16.7)	1.86 (1.29–2.67)	10.6 (8.5–13.2)	1.38 (0.95–2.02)	7.9 (6.1–10.2)
No	24.1 (21.9–26.4)	2.86 (2.30–3.55)	17.2 (15.3–19.3)	1.88 (1.50–2.35)	18.1 (16.2–20.2)	1.99 (1.60–2.49)	10.0 (8.5–11.7)

CAR = continuous abstinence rate; CI = confidence interval; OR = odds ratio; NRT = nicotine replacement therapy (transdermal nicotine patch); HADS = Hospital Anxiety and Depression Scale; C‐SSRS = Columbia‐Suicide Severity Rating Scale; BPAQ = Buss–Perry Aggression Questionnaire.

Results indicate that ORs for treatment versus placebo comparisons were consistent across different values of covariates, with larger deviations limited to strata with small sample sizes.

## Discussion

The findings of these analyses are consistent with prior evidence showing lower chances of success of quit attempts in smokers with higher levels of cigarette addiction, poorer mental health and younger age, making it clear that these associations are independent of each other and independent of other putative covariates. Pharmacotherapy did not influence this vulnerability, but rather improved abstinence rates across the board. Overall, varenicline almost tripled—and bupropion and NRT almost doubled—the odds of quitting versus placebo at 6 months, and the treatment × covariate interactions were not significant. These findings also provide the clearest evidence to date that younger age of starting to smoke and US origin are associated with lower success rates of stopping regardless of treatment, even after controlling for other covariates. The findings also provide suggestive evidence, once other covariates have been adjusted for, that smokers with higher BMI may have a greater likelihood of success of stopping. Those who have used any form of NRT in the past may have a lower likelihood of success overall. However, prior use of a given pharmacotherapy did not appear to reduce the impact of that or another pharmacotherapy in a given quit attempt.

The fact that varenicline, bupropion and NRT were all effective in smokers with mental health problems, assessed with a number of variables (e.g. diagnostic history, HADS, use of psychotropic medication), and their relative efficacy was similar to that in smokers without a psychiatric history, is clinically important. Part of the reluctance of clinicians to treat cigarette addiction in patients with mental health problems may stem from a lack of confidence that it would be helpful. This concern can now be dispelled, even in smokers with psychotic disorders.

The apparent absence of interactions between treatment type and smokers’ baseline characteristics, despite the large sample size and wide range of characteristics assessed, suggests that clinicians should always encourage smokers to use what is, on average, the most effective treatment option rather than attempting to match smokers to treatments by any of the variables assessed. In particular, the finding that pharmacotherapy efficacy did not appear to be affected by prior use of that treatment (Table 3) has important implications for clinical practice, but it seems to conflict with an observational study which found that smokers who switched to a different pharmacotherapy in a subsequent quit attempt were more likely to succeed in the short term than those who persisted with the one used previously 32. That was a correlational study and involved smokers making a decision to switch rather than being randomized to a medicine they may or may not have used previously. EAGLES shows that such a switch might not be necessary and in fact might be counter‐productive if it means switching from a more to less effective pharmacotherapy.

However, of concern is the finding that past use of NRT in the stepwise model and past use of any of the treatments in the simple model were associated with lower success rates overall (Table 2). In the case of NRT, lower success rate was not accounted for by cigarette addiction or other covariates as measured. It remains possible, however, that it reflects the impact of unmeasured residual confounding. This could be an aspect of cigarette addiction that is not captured adequately by the FTCD or some other factor. In the context of findings in the electronic cigarette literature—that past use of these devices is associated with reduced rates of smoking cessation but not reduced quit attempt rates 33—the present findings raise the possibility that use of nicotine in a form other than smoking may, for an as yet unknown reason, reduce the ability of smokers to stop smoking. Given the widespread use of nicotine products by smokers, this association is worthy of further research.

The lower success rates in US smokers compared with those in other countries is in line with informal cross‐study comparisons. The findings tend to support the view that smokers in countries such as the United States have reached a point in the tobacco epidemic where the relatively low proportion of people who continue to smoke, despite strong cultural pressures not to, have particular characteristics that make it more difficult for them to stop. The lower success rate in the United States versus other smokers was not attenuated in the stepwise model, suggesting that other factors may explain the country differences.

The lower success rate among black smokers has been observed in other studies in the United States 34. Because ethnic difference remained in the stepwise model suggests that factors other than cigarette addiction and other measured vulnerabilities are responsible. This, too, is an important area of future study.

No difference was observed between male and female smokers in terms of success rates for smoking cessation. This observation is in accordance with population‐level studies 4, but is at variance with some clinical studies 16. It is unclear why some studies find sex differences while others do not. It seems unlikely to be due to sampling variation. To our knowledge, no attempt has been made to identify moderators of potential sex differences. Doing so could provide important insights into cigarette addiction and would be worthwhile.

One surprising result was the emergence of BMI as being associated positively with success in the stepwise model. The effect size was small, and it is possible that this represents a Type 1 error. However, it suggests that future studies examine this association to clarify any relationship.

These analyses had a number of limitations. First, the analyses were not pre‐planned or registered. While, during the development of the EAGLES trial, it was intended to examine predictors of success and treatment moderators, such analyses are often not performed following RCTs, which raises the risk of reporting bias. There is a movement to require pre‐specification of all hypothesis‐testing studies, but currently it is not standard practice. Until it is, and until there is ready access to such registrations, all findings from correlational analyses must be viewed with caution.

Another limitation is that even though EAGLES was a large study, the sample sizes in some strata were relatively small. It therefore remains possible that these analyses did not detect predictive or moderator relationships that were present. It is unlikely that a smoking cessation pharmacotherapy evaluation of this size and scope will be conducted again in the next decade or so. This means that identifying such relationships will rely upon aggregating across studies. This, in turn, means that researchers must report their findings in a way that will permit aggregation, or preferably make their data available for synthesis.

A third limitation relates to the categories used for some of the covariates; thus, it was necessary to pool all non‐US smokers. Clearly, there will be considerable heterogeneity among this group, which must temper conclusions about the US versus non‐US comparison. Additionally, the psychiatric disorder classification was relatively broad, and it may be that there is heterogeneity within the categories used.

In conclusion, the EAGLES study confirmed that younger age, higher level of cigarette addiction and psychiatric disorder independently predict failure to achieve continuous abstinence. It found that lower age of starting to smoke, use of psychotropic medication, having used NRT previously, being in the United States (versus non‐USA) and being black (versus white) were also associated independently with lack of success in stopping smoking and that high BMI appeared to be predictive of success at stopping. Importantly, it did not find a significant treatment moderation effect of these or other measured covariates. The findings raise important issues for future research, but from a clinical viewpoint they provide unique new evidence on the benefit of treating cigarette addiction with pharmacotherapy regardless of smokers’ mental health status or other demographic and smoking history characteristics.

## Clinical trial registration

Clinical trial registration no. NCT01456936 (https://clinicaltrials.gov/ct2/show/NCT01456936).

## Declaration of interests

R.W. reports grants from Johnson & Johnson and Pfizer and personal fees for consulting and/or advisory board services to GlaxoSmithKline, Johnson & Johnson and Pfizer. R.W.'s writing of this paper was supported, in part, by Cancer Research United Kingdom. A.E.E. reports grants from Forum Pharmaceuticals and Pfizer and personal fees for advisory board services from Pfizer and Reckitt Benckiser. A.E.E.'s writing of the paper was supported by a National Institute on Drug Abuse Career Award in Patient‐Oriented Research, K24 DA030443. N.L.B. reports consulting and/or advisory board services to GlaxoSmithKline and Pfizer and is a paid expert witness in litigation against tobacco companies. C.R., T.M., D.L. and L.S.A. are employees and stockholders of Pfizer. A.K. is a PAREXEL employee working on behalf of GlaxoSmithKline. M.C.M. reports no competing interests. R.M.A. reports grants from Alkermes and Pfizer and consulting and/or advisory board services to Arena Pharmaceuticals, Cerecor and Pfizer. R.M.A.'s writing of this paper was supported, in part, by National Institute on Alcohol Abuse and Alcoholism Grants U01 AA013641 and R01 AA019720 and National Institute on Drug Abuse/Veterans Affairs Cooperative Studies numbers 1032 and 1033. The opinions expressed in this paper are those of the authors and do not necessarily reflect the views of their employers.

## Supporting information


**Table S1** Subject enrollment* and site presence by country/region, by treatment.

**Table S2** Collection of smokers’ characteristics using the patient report form completed by investigators.
