# Supplementary material for: Factors associated with the efficacy of smoking cessation treatments and predictors of smoking abstinence in EAGLES
Source: Addiction. 2018 Mar 30;113(8):1507–16. doi: 10.1111/add.14208 (PMC6055735; doi:10.1111/add.14208)
Supplement: Supplementary file 1 — Table S1 Subject enrollment* and site presence by country/region, by treatment. [file ADD-113-1507-s001.docx]

**Table S1** Subject enrolment* and site presence by country/region, by treatment

|  |  | *Varenicline*  *(n = 2016)* | | *Bupropion*  *(n = 2006)* | | *NRT*  *(n = 2022)* | | *Placebo*  *(n = 2014)* | | All  (*N = 8058*) |
| --- | --- | --- | --- | --- | --- | --- | --- | --- | --- | --- |
| Region | Site No. | NPC | PC | NPC | PC | NPC | PC | NPC | PC |  |
| *United States* | | | | | | | | | | |
| United States | 1001 | 11 | 4 | 6 | 9 | 8 | 7 | 11 | 12 | 68 |
|  | 1002 | 11 | 13 | 13 | 12 | 14 | 21 | 11 | 17 | 112 |
|  | 1003 | 25 | 2 | 36 | 3 | 32 | 4 | 31 | 4 | 137 |
|  | 1004 | 7 | 27 | 9 | 29 | 12 | 27 | 4 | 32 | 147 |
|  | 1005 | 8 | 3 | 6 | 2 | 16 | 4 | 12 | 4 | 55 |
|  | 1007 | 5 | 3 | 6 | 1 | 4 | 3 | 5 | 6 | 33 |
|  | 1009 | 11 | 14 | 4 | 13 | 3 | 8 | 6 | 11 | 70 |
|  | 1010 | 4 | 8 | 6 | 5 | 8 | 6 | 5 | 10 | 52 |
|  | 1011 | 12 | 3 | 4 | 5 | 7 | 4 | 13 | 7 | 55 |
|  | 1014 | 11 | 4 | 12 | 3 | 10 | 5 | 9 | 2 | 56 |
|  | 1016 | 10 | 10 | 11 | 15 | 11 | 8 | 6 | 11 | 82 |
|  | 1018 | 1 | 0 | 0 | 1 | 1 | 0 | 0 | 0 | 3 |
|  | 1019 | 15 | 14 | 26 | 13 | 22 | 18 | 16 | 8 | 132 |
|  | 1024 | 7 | 0 | 2 | 0 | 5 | 0 | 4 | 0 | 18 |
|  | 1025 | 15 | 2 | 14 | 3 | 19 | 3 | 12 | 2 | 70 |
|  | 1028 | 4 | 2 | 7 | 2 | 5 | 4 | 7 | 1 | 32 |
|  | 1029 | 8 | 0 | 12 | 4 | 9 | 0 | 19 | 3 | 55 |
|  | 1031 | 1 | 0 | 3 | 0 | 0 | 0 | 2 | 0 | 6 |
|  | 1034 | 4 | 9 | 3 | 4 | 5 | 3 | 5 | 6 | 39 |
|  | 1038 | 2 | 2 | 1 | 4 | 0 | 3 | 3 | 5 | 20 |
|  | 1040 | 0 | 13 | 0 | 11 | 0 | 6 | 0 | 13 | 43 |
|  | 1041 | 1 | 6 | 1 | 4 | 2 | 7 | 4 | 2 | 27 |
|  | 1045 | 3 | 1 | 2 | 1 | 5 | 0 | 4 | 0 | 16 |
|  | 1055 | 3 | 27 | 4 | 29 | 0 | 22 | 2 | 24 | 111 |
|  | 1056 | 7 | 7 | 6 | 8 | 2 | 9 | 8 | 5 | 52 |
|  | 1057 | 29 | 31 | 42 | 27 | 44 | 26 | 31 | 25 | 255 |
|  | 1059 | 16 | 32 | 19 | 45 | 15 | 40 | 18 | 30 | 215 |
|  | 1062 | 0 | 5 | 1 | 4 | 1 | 4 | 2 | 4 | 21 |
|  | 1063 | 19 | 2 | 15 | 5 | 25 | 4 | 20 | 4 | 94 |
|  | 1067 | 19 | 24 | 14 | 22 | 18 | 21 | 12 | 27 | 157 |
|  | 1071 | 0 | 1 | 2 | 1 | 2 | 0 | 2 | 1 | 9 |
|  | 1072 | 5 | 4 | 2 | 2 | 0 | 1 | 3 | 1 | 18 |
|  | 1074 | 2 | 8 | 3 | 10 | 3 | 14 | 2 | 7 | 49 |
|  | 1076 | 3 | 1 | 2 | 1 | 0 | 3 | 3 | 1 | 14 |
|  | 1077 | 4 | 6 | 8 | 1 | 5 | 0 | 6 | 1 | 31 |
|  | 1080 | 22 | 14 | 14 | 22 | 19 | 22 | 13 | 16 | 142 |
|  | 1081 | 23 | 9 | 24 | 12 | 25 | 15 | 28 | 16 | 152 |
|  | 1083 | 7 | 6 | 11 | 4 | 8 | 5 | 8 | 5 | 54 |
|  | 1086 | 0 | 2 | 2 | 4 | 2 | 0 | 2 | 1 | 13 |
|  | 1094 | 11 | 14 | 3 | 19 | 8 | 16 | 5 | 24 | 100 |
|  | 1096 | 0 | 9 | 0 | 8 | 0 | 5 | 0 | 9 | 31 |
|  | 1098 | 14 | 1 | 21 | 10 | 21 | 6 | 15 | 5 | 93 |
|  | 1099 | 2 | 12 | 1 | 6 | 7 | 6 | 4 | 3 | 41 |
|  | 1102 | 3 | 0 | 1 | 0 | 1 | 0 | 1 | 1 | 7 |
|  | 1106 | 8 | 23 | 13 | 16 | 5 | 13 | 10 | 14 | 102 |
|  | 1109 | 2 | 7 | 2 | 12 | 3 | 7 | 4 | 5 | 42 |
|  | 1110 | 25 | 12 | 15 | 7 | 23 | 12 | 32 | 14 | 140 |
|  | 1111 | 9 | 6 | 5 | 1 | 5 | 1 | 5 | 3 | 35 |
|  | 1112 | 10 | 19 | 8 | 8 | 5 | 9 | 6 | 13 | 78 |
|  | 1174 | 27 | 13 | 21 | 15 | 16 | 15 | 20 | 16 | 143 |
|  | 1220 | 18 | 28 | 23 | 35 | 15 | 30 | 18 | 29 | 196 |
|  | 1247 | 0 | 11 | 0 | 12 | 0 | 16 | 0 | 15 | 54 |
|  | 1248 | 0 | 24 | 0 | 20 | 0 | 18 | 0 | 29 | 91 |
|  | 1249 | 0 | 8 | 0 | 4 | 0 | 8 | 0 | 8 | 28 |
|  | 1251 | 0 | 17 | 0 | 17 | 0 | 23 | 0 | 14 | 71 |
|  | 1253 | 0 | 33 | 0 | 26 | 0 | 22 | 0 | 22 | 103 |
|  | 1259 | 0 | 0 | 0 | 1 | 0 | 0 | 0 | 0 | 1 |
|  | 1260 | 0 | 2 | 0 | 5 | 0 | 4 | 0 | 6 | 17 |
|  | 1261 | 0 | 17 | 0 | 8 | 0 | 18 | 0 | 8 | 51 |
|  | 1262 | 0 | 1 | 0 | 1 | 0 | 3 | 0 | 6 | 11 |
|  | 1263 | 0 | 5 | 0 | 3 | 0 | 7 | 0 | 2 | 17 |
|  | 1264 | 0 | 2 | 0 | 2 | 0 | 3 | 0 | 1 | 8 |
|  | 1266 | 0 | 1 | 0 | 5 | 0 | 2 | 0 | 4 | 12 |
|  | 1267 | 0 | 6 | 0 | 4 | 0 | 4 | 0 | 6 | 20 |
| *Western Europe and other countries* | | | | | | | | | | |
| Australia | 1196 | 0 | 0 | 0 | 1 | 0 | 1 | 2 | 1 | 5 |
|  | 1234 | 4 | 4 | 6 | 9 | 11 | 3 | 8 | 5 | 50 |
| Canada | 1047 | 7 | 1 | 6 | 1 | 5 | 1 | 3 | 3 | 27 |
|  | 1048 | 1 | 2 | 7 | 2 | 2 | 6 | 3 | 2 | 25 |
|  | 1049 | 7 | 6 | 9 | 2 | 7 | 3 | 5 | 6 | 45 |
|  | 1050 | 0 | 3 | 0 | 0 | 0 | 4 | 0 | 2 | 9 |
|  | 1051 | 10 | 13 | 5 | 9 | 9 | 12 | 10 | 10 | 78 |
|  | 1052 | 12 | 13 | 7 | 14 | 9 | 13 | 12 | 13 | 93 |
| Denmark | 1199 | 18 | 1 | 21 | 2 | 12 | 2 | 19 | 1 | 76 |
|  | 1242 | 9 | 0 | 8 | 0 | 12 | 0 | 8 | 0 | 37 |
| Finland | 1204 | 7 | 16 | 9 | 15 | 7 | 20 | 10 | 18 | 102 |
|  | 1208 | 11 | 10 | 15 | 3 | 5 | 19 | 6 | 12 | 81 |
|  | 1209 | 3 | 11 | 2 | 4 | 4 | 14 | 5 | 5 | 48 |
|  | 1210 | 2 | 11 | 6 | 8 | 7 | 6 | 5 | 8 | 53 |
|  | 1211 | 5 | 10 | 7 | 13 | 7 | 21 | 7 | 13 | 83 |
|  | 1218 | 12 | 24 | 15 | 24 | 9 | 18 | 12 | 20 | 134 |
| Germany | 1087 | 6 | 2 | 6 | 1 | 4 | 1 | 2 | 1 | 23 |
|  | 1088 | 33 | 40 | 22 | 47 | 23 | 49 | 21 | 50 | 285 |
|  | 1089 | 2 | 7 | 1 | 1 | 7 | 2 | 2 | 5 | 27 |
|  | 1090 | 4 | 10 | 11 | 9 | 10 | 12 | 5 | 5 | 66 |
|  | 1126 | 7 | 22 | 8 | 25 | 8 | 19 | 6 | 15 | 110 |
|  | 1144 | 29 | 8 | 29 | 10 | 28 | 6 | 23 | 10 | 143 |
|  | 1154 | 20 | 31 | 23 | 32 | 28 | 19 | 32 | 37 | 222 |
| New Zealand | 1198 | 12 | 10 | 15 | 22 | 20 | 9 | 20 | 17 | 125 |
| South Africa | 1129 | 11 | 7 | 2 | 5 | 7 | 7 | 7 | 8 | 54 |
|  | 1130 | 7 | 0 | 10 | 0 | 6 | 0 | 16 | 1 | 40 |
|  | 1131 | 13 | 1 | 10 | 1 | 11 | 1 | 9 | 0 | 46 |
|  | 1132 | 7 | 0 | 9 | 1 | 7 | 1 | 9 | 0 | 34 |
|  | 1134 | 2 | 0 | 6 | 0 | 3 | 0 | 8 | 0 | 19 |
|  | 1135 | 3 | 3 | 5 | 1 | 9 | 0 | 6 | 1 | 28 |
|  | 1140 | 5 | 9 | 0 | 9 | 4 | 8 | 2 | 5 | 42 |
|  | 1147 | 0 | 1 | 1 | 0 | 0 | 0 | 0 | 0 | 2 |
|  | 1239 | 8 | 0 | 10 | 1 | 6 | 0 | 5 | 0 | 30 |
| Spain | 1161 | 18 | 10 | 10 | 10 | 18 | 14 | 17 | 6 | 103 |
|  | 1162 | 0 | 4 | 1 | 5 | 1 | 5 | 3 | 9 | 28 |
|  | 1163 | 5 | 2 | 2 | 1 | 1 | 5 | 2 | 2 | 20 |
|  | 1164 | 8 | 3 | 6 | 3 | 8 | 2 | 6 | 4 | 40 |
|  | 1165 | 14 | 0 | 10 | 0 | 7 | 0 | 10 | 1 | 42 |
|  | 1246 | 0 | 2 | 0 | 1 | 0 | 0 | 0 | 1 | 4 |
| *Eastern Europe* | | | | | | | | | | |
| Bulgaria | 1177 | 2 | 6 | 2 | 8 | 4 | 14 | 4 | 9 | 49 |
|  | 1178 | 7 | 5 | 13 | 4 | 6 | 2 | 12 | 1 | 50 |
|  | 1179 | 17 | 4 | 13 | 8 | 9 | 6 | 10 | 8 | 75 |
|  | 1181 | 16 | 6 | 7 | 8 | 10 | 9 | 10 | 9 | 75 |
|  | 1182 | 8 | 6 | 11 | 7 | 5 | 6 | 3 | 3 | 49 |
|  | 1183 | 5 | 15 | 5 | 4 | 11 | 9 | 8 | 12 | 69 |
|  | 1207 | 4 | 0 | 1 | 0 | 5 | 0 | 4 | 0 | 14 |
|  | 1212 | 3 | 11 | 2 | 10 | 3 | 10 | 1 | 10 | 50 |
|  | 1232 | 3 | 3 | 4 | 2 | 7 | 3 | 9 | 3 | 34 |
|  | 1241 | 0 | 3 | 3 | 2 | 7 | 1 | 6 | 3 | 25 |
| Russian Federation | 1153 | 11 | 0 | 8 | 0 | 6 | 0 | 6 | 0 | 31 |
|  | 1157 | 0 | 4 | 0 | 5 | 0 | 3 | 0 | 4 | 16 |
|  | 1158 | 3 | 0 | 7 | 1 | 4 | 1 | 1 | 0 | 17 |
|  | 1160 | 4 | 0 | 4 | 0 | 9 | 0 | 3 | 0 | 20 |
|  | 1187 | 1 | 2 | 0 | 2 | 0 | 2 | 1 | 1 | 9 |
|  | 1255 | 0 | 2 | 0 | 2 | 0 | 2 | 0 | 2 | 8 |
|  | 1256 | 0 | 1 | 0 | 3 | 0 | 3 | 0 | 1 | 8 |
|  | 1257 | 0 | 0 | 0 | 0 | 0 | 1 | 0 | 0 | 1 |
|  | 1258 | 0 | 5 | 0 | 5 | 0 | 4 | 0 | 2 | 16 |
| Slovakia | 1213 | 3 | 3 | 2 | 1 | 4 | 2 | 2 | 2 | 19 |
|  | 1214 | 6 | 2 | 9 | 1 | 2 | 0 | 9 | 5 | 34 |
|  | 1215 | 1 | 6 | 3 | 9 | 3 | 3 | 3 | 6 | 34 |
|  | 1216 | 3 | 7 | 4 | 6 | 2 | 6 | 1 | 7 | 36 |
|  | 1217 | 14 | 3 | 14 | 4 | 15 | 6 | 18 | 5 | 79 |
| *South and Middle America* | | | | | | | | | | |
| Argentina | 1108 | 38 | 21 | 41 | 19 | 44 | 25 | 46 | 19 | 253 |
|  | 1139 | 18 | 6 | 9 | 7 | 10 | 3 | 12 | 11 | 76 |
| Brazil | 1119 | 1 | 2 | 0 | 0 | 0 | 1 | 0 | 0 | 4 |
|  | 1120 | 0 | 0 | 0 | 0 | 2 | 0 | 0 | 0 | 2 |
|  | 1121 | 1 | 2 | 1 | 5 | 1 | 1 | 1 | 1 | 13 |
|  | 1122 | 0 | 0 | 1 | 0 | 0 | 0 | 1 | 0 | 2 |
| Chile | 1172 | 4 | 2 | 3 | 1 | 0 | 1 | 4 | 0 | 15 |
|  | 1202 | 0 | 0 | 2 | 0 | 0 | 0 | 0 | 0 | 2 |
| Mexico | 1171 | 0 | 0 | 0 | 0 | 1 | 0 | 0 | 0 | 1 |
|  | 1222 | 3 | 0 | 0 | 0 | 3 | 0 | 0 | 0 | 6 |
|  | 1228 | 14 | 11 | 22 | 15 | 26 | 14 | 18 | 13 | 133 |
|  | 1240 | 14 | 1 | 12 | 0 | 9 | 0 | 11 | 0 | 47 |

NRT = nicotine replacement therapy (transdermal nicotine patch); NPC, non-psychiatric cohort; PC, psychiatric cohort. *All-treated population in the EAGLES study.
