# Supplementary material for: Factors associated with the efficacy of smoking cessation treatments and predictors of smoking abstinence in EAGLES
Source: Addiction. 2018 Mar 30;113(8):1507–16. doi: 10.1111/add.14208 (PMC6055735; doi:10.1111/add.14208)
Supplement: Supplementary file 2 — Table S2 Collection of smokers’ characteristics using the patient report form completed by investigators. [file ADD-113-1507-s002.docx]

**Table S2** Collection of smokers’ characteristics using the patient report form completed by investigators

| *Baseline covariate as listed in Table 1 ‘Sample characteristics’* | *Investigator report form descriptor* | *Question posed/data collected* |
| --- | --- | --- |
| Age, years | Date of birth | Date of Birth (dd-MMM-yyyy) |
| Age of starting smoking, years | Smoking history – Lifetime | At what age did the subject start smoking? |
| Female | Sex | Response options:   - Male - Female   If subject is transsexual, record the original sex of the subject. Record the surgical procedure in medical history section and the hormone treatment in concomitant treatment section |
| Ethnic group | Race | Response options:   - White - Black - Asian - Other   If Other is selected, specify the race in the space provided. Do not specify a race in the comment space if you select White, Black, or Asian |
| Country | N/A | Based on study site |
| BMI, kg/m^2^ | N/A – Calculated based on height and  weight as recorded on report form | - Height (inches or cm) - Weight (lb or kg) |
| History of psychiatric diagnosis | Structured Clinical Interview for  DSM-IV (SCID) | - Not done, *or* - Recorded based on SCID with only one primary diagnostic permitted on the report form: - Mood disorders - Anxiety disorders - Schizophrenia and other psychotic disorders - Personality disorders |
| Alcohol/substance abuse disorder history | Structured Clinical Interview for  DSM-IV (SCID) | - Not done, *or* - Recorded based on SCID (Substance abuse disorders), permitted as co-morbid diagnostic only on the report form |
| HADS anxiety score | Hospital Anxiety and Depression Scale | - Not done, *or* - Recorded as per scale |
| HADS depression score | Hospital Anxiety and Depression Scale | - Not done, *or* - Recorded as per scale |
| C-SSRS prior suicidal ideation  and/or behaviour | Columbia Suicide-Severity Rating  Scale – Lifetime | - Not done, *or* - Recorded as per scale |
| BPAQ aggression score | Aggression questionnaire | - Not done, *or* - Recorded as per questionnaire |
| Baseline psychotropic medication | Previous and concomitant psychotropic drug treatment – 12 months | - None, *or* - Drug name - Reason - Duration (start/stop date, or ongoing) - Total daily dose (units) |
| FTCD | Fagerström Test for Nicotine dependence | - Not done, *or* - Recorded as per questionnaire |
| Prior varenicline use  Prior bupropion use  Prior any NRT use | Smoking history – Lifetime | What are the type and number of lifetime serious quit attempts? (serious quit attempt = more than 24 hours)   - None - Nicotine patch - Nicotine gum - Nicotine inhaler - Nicotine spray - Nicotine Lozenge - Bupropion HCL - Hypnosis - Cold Turkey - Counselling/self-help - Varenicline - Other (specify) |

NRT = nicotine replacement therapy (transdermal nicotine patch). N/A = not applicable. BMI = body mass index. HADS = Hospital Anxiety and Depression Scale. C-SSRS = Columbia-Suicide Severity Rating Scale. BPAQ = Buss-Perry Aggression Questionnaire. FTCD = Fagerström Test for Cigarette Dependence.
